# Supplementary material for: Phosphorus-Mediated Transition from Vegetative to Reproductive Growth in Dwarf Coconut (Cocos nucifera L.)
Source: Int J Mol Sci. 2024 Nov 9;25(22):12040. doi: 10.3390/ijms252212040 (PMC11593421; doi:10.3390/ijms252212040)
Supplement: Supplementary file 1 [file ijms-25-12040-s001.zip › ijms-3285092-supplementary.pdf]

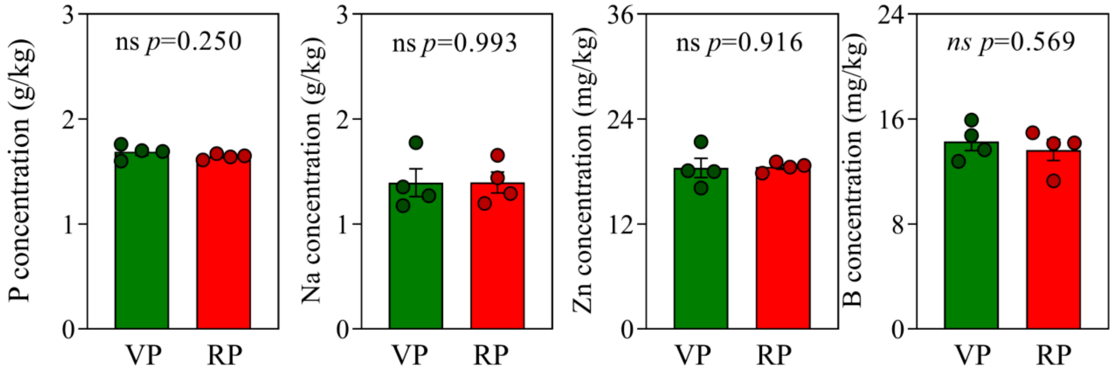

Figure S1: Analysis of nutrient in coconut leave at different phases

VP, Vegetative phase; RP, Reproductive phase. Each treatment was conducted with four biological replicates. ns, no statistical difference. Each treatment was performed with four biological repetitions, the dots represent the biological replicates, with green representing VP coconuts and red representing RP coconuts. ns depicts insignificance.

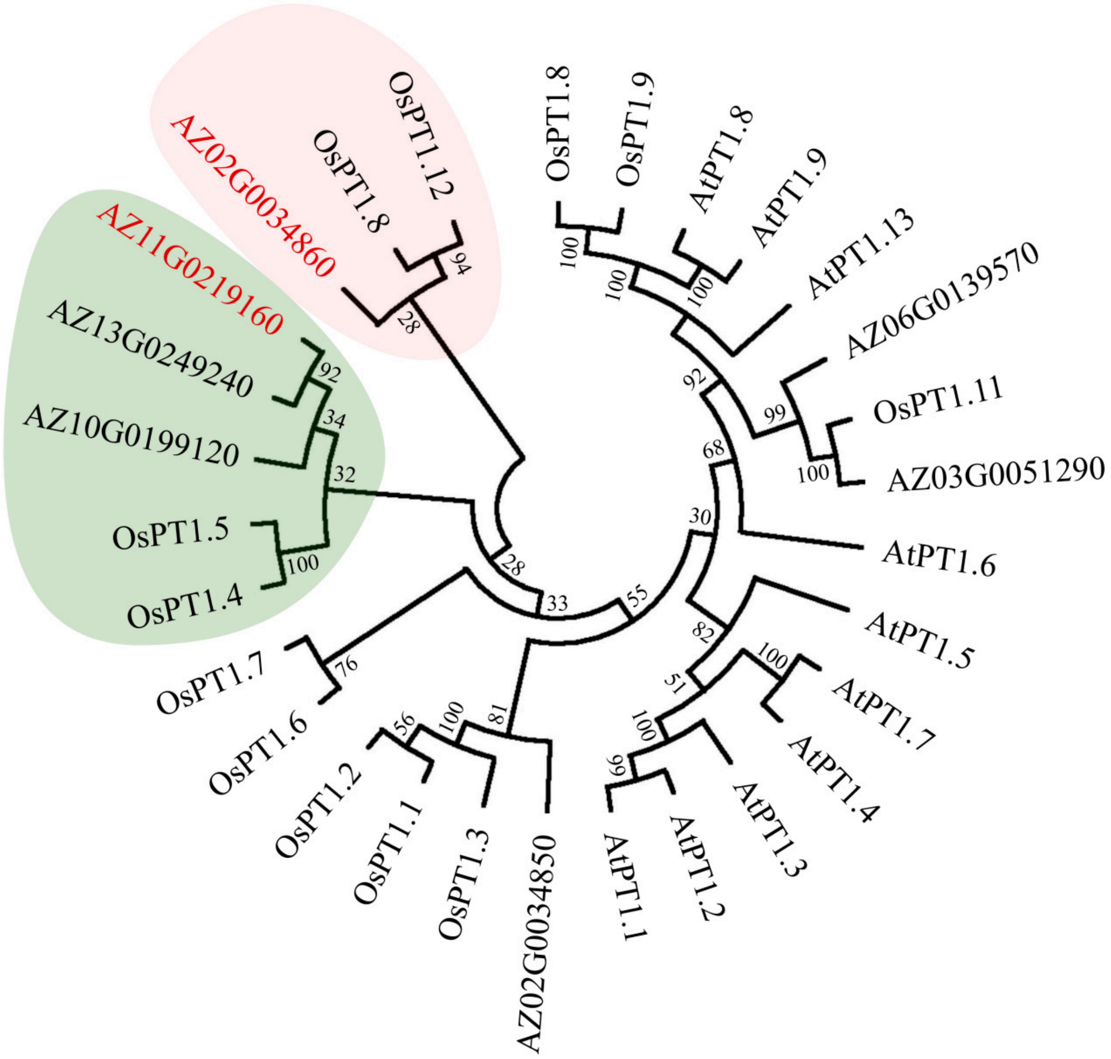

Figure S2: Phylogenetic analysis of the *PT1* family members in coconut, rice, and *Arabidopsis*

The phylogenetic tree illustrates the results of the co-evolution analysis of the *PT1* gene family in coconut, rice, and *Arabidopsis*. The numbers on the evolutionary tree represent the confidence levels of the phylogenetic branching.

Table S1: Bioinformatics characteristics of the *PT1* family members in coconut.

NO.	Gene	Protein Length(aa)	Chr	MW (kDa)	TM	pI	Reliability(%): Predicted Location
1	AZ03G0051290	518	3	57.37	11	8.75	65.2%: Plasma membrane
2	AZ06G0139570	533	6	57.92	11	8.79	47.8%: Plasma membrane
3	AZ13G0249240	519	13	56.81	8	8.75	52.2%: Plasma membrane
4	AZ02G0034860	533	2	58.15	11	8.89	52.2%: Plasma membrane
5	AZ10G0199120	536	10	58.76	11	8.89	55.6%: Plasma membrane
6	AZ11G0219160	540	11	58.94	12	8.59	33.3%: Plasma membrane
7	AZ02G0034850	528	2	58.07	11	8.59	33.3%: Plasma membrane

Figure S3: Analysis of the coconut and Rice PT1 Protein Sequences

Red asterisks (*) indicate completely conserved amino acid sites, meaning that the amino acids at these positions are identical in all sequences. Black hyphens (-) indicate non-conserved amino acid sites, meaning that the amino acids at these positions are different across various sequences.

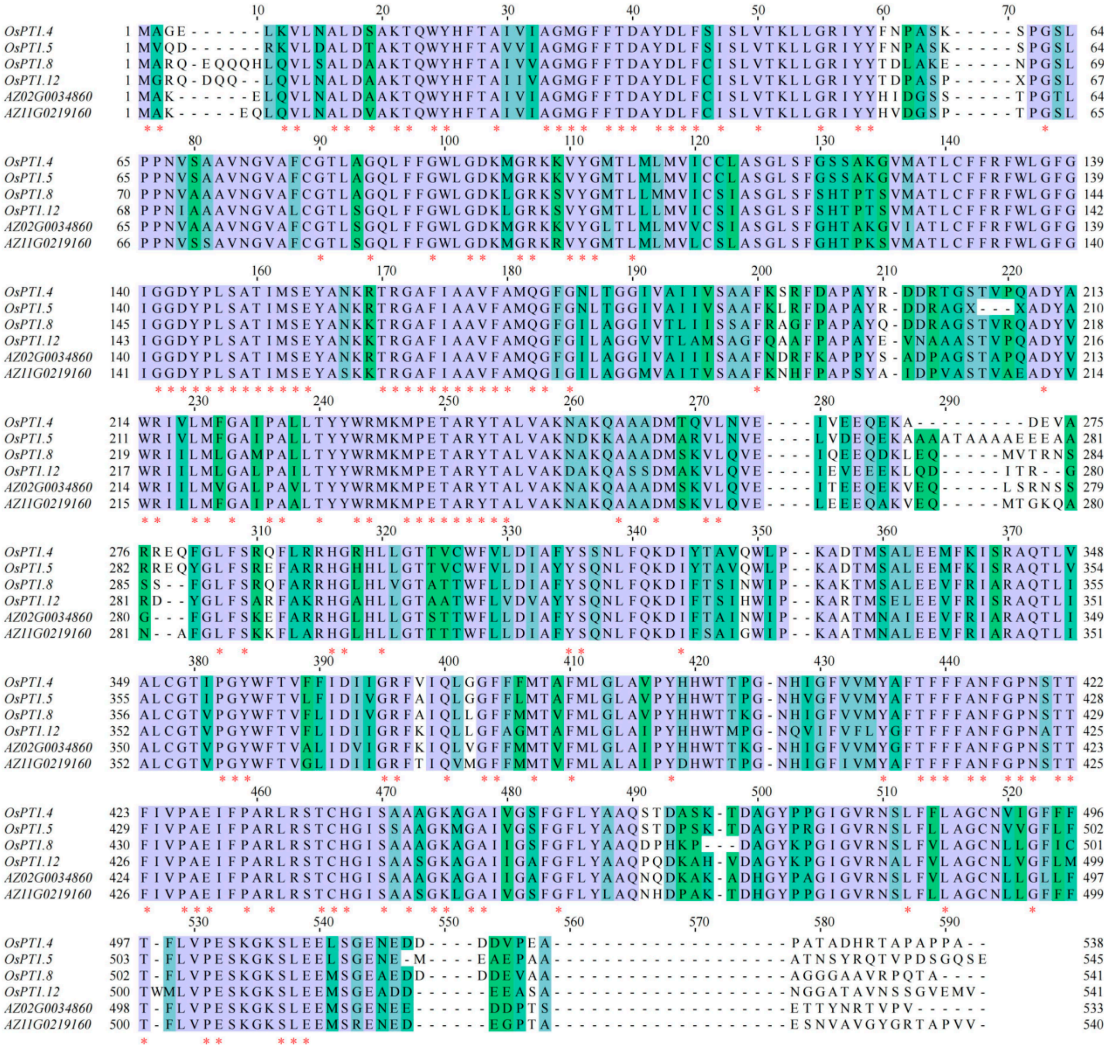

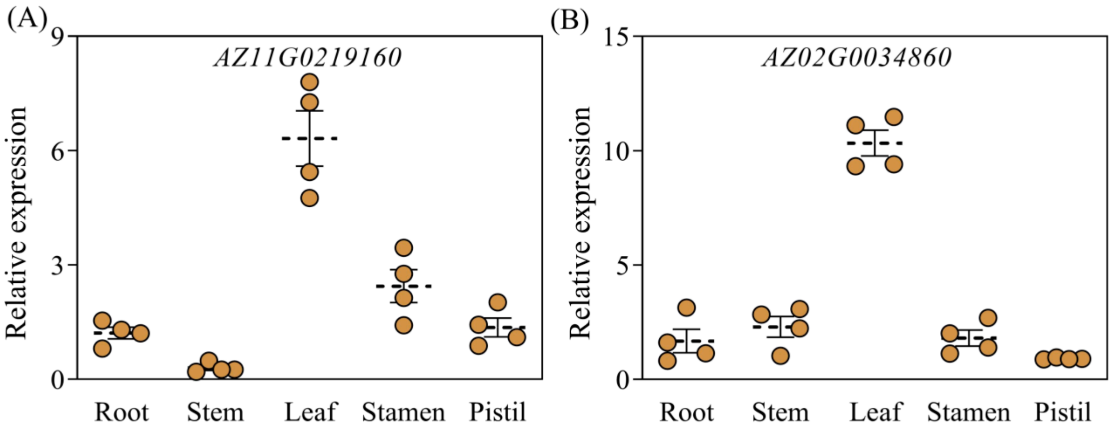

Figure S4: Tissue expression patterns of key *PT1s* in coconut

(A) Quantitative expression levels of the coconut phosphate transporter gene *AZ11G0219160* in the roots, stems, leaves, stamen and pistil of Wenye 2 during the reproductive phases. (B) Quantitative expression levels of the coconut phosphate transporter gene *AZ02G0034860* in the roots, stems, leaves, stamen and pistil of Wenye 2 during the reproductive phases. Each treatment was performed with four biological repetitions, the dots represent the biological replicates.

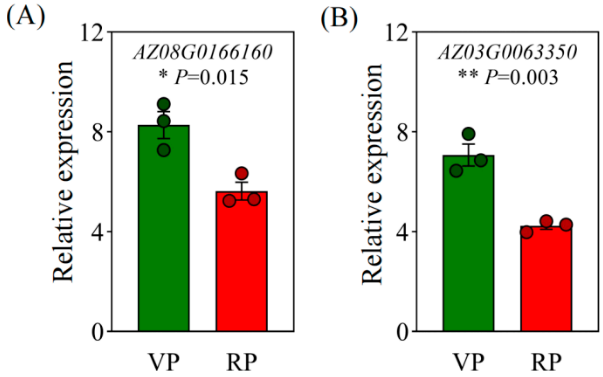

Figure S5: FPKM of genes involved in plant hormone synthesis in VP and RP of coconut root

(A) *AZ08G0166160* gene encodes the gibberellin 2 beta-dioxygenase enzyme G2OX3, which is involved in the degradation of gibberellins. (B) *AZ03G0063350* gene encodes the cytokinin dehydrogenase enzyme CKX5, which is involved in the degradation of cytokinin. Each treatment was performed with three biological repetitions, the dots represent the biological replicates, with green representing VP coconuts and red representing RP coconuts. * depicts significant difference at the $0.01 < p \leq 0.05$ level; ** depicts significant difference at the $0.001 < p \leq 0.01$ level.

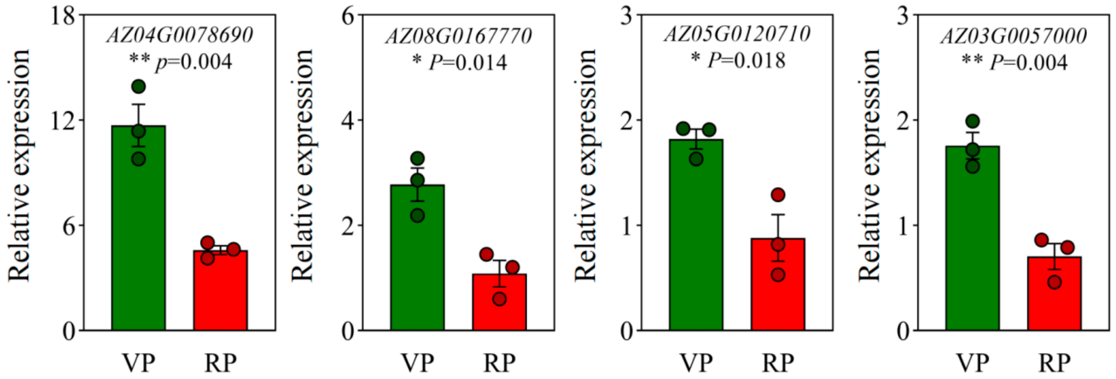

Figure S6: FPKM of *PINs* genes in VP and RP of coconut root

Each treatment was performed with three biological repetitions, the dots represent the biological replicates, with green representing VP coconuts and red representing RP coconuts. * depicts significant difference at the $0.01 < p \leq 0.05$ level; ** depicts significant difference at the $0.001 < p \leq 0.01$ level.

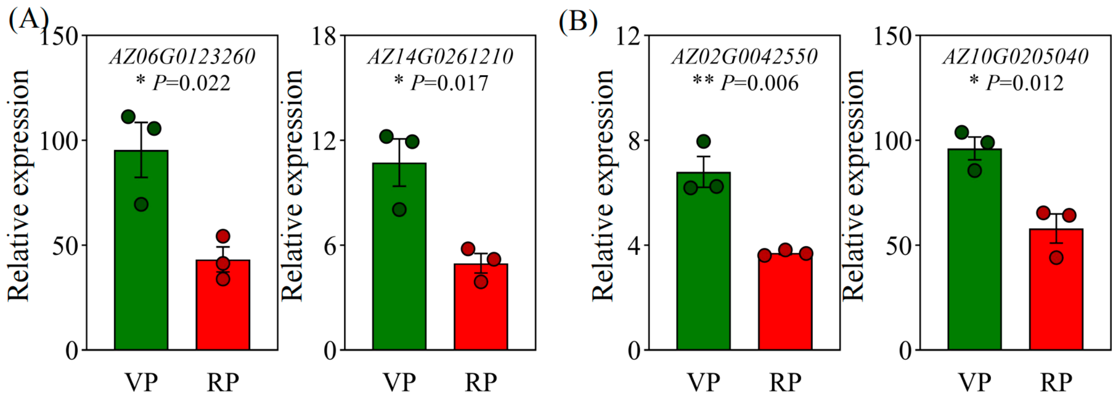

Figure S7: FPKM of *LAXs* and *PYLs* genes in VP and RP of coconut root

(A) *AZ06G0123260* and *AZ14G0261210* gene encodes the auxin influx carrier LAXs. (B) *AZ02G0042550* and *AZ10G0205040* gene encodes the abscisic acid receptor PYR/PYLs. Each treatment was performed with three biological repetitions, the dots represent the biological replicates, with green representing VP coconuts and red representing RP coconuts. * depicts significant difference at the $0.01 < p \leq 0.05$ level; ** depicts significant difference at the $0.001 < p \leq 0.01$ level.

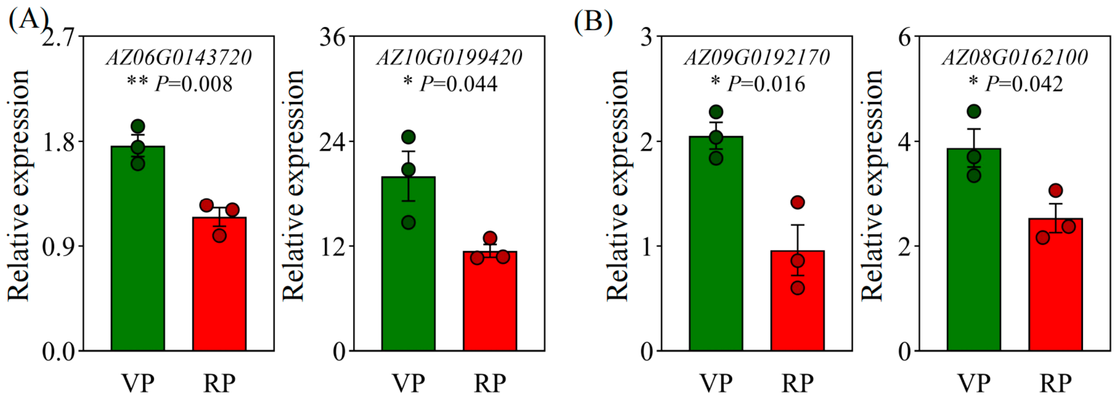

Figure S8: FPKM of *ETRs* and *BRLs* genes in VP and RP of coconut root
(A) *AZ06G0143720* and *AZ10G0199420* gene encodes the ethylene receptor ETR2. (B) *AZ09G0192170* and *AZ08G0162100* gene encodes the brassinosteroid LRR receptor kinase BRL2. Each treatment was performed with three biological repetitions, the dots represent the biological replicates, with green representing VP coconuts and red representing RP coconuts. * depicts significant difference at the $0.01 < p \leq 0.05$ level; ** depicts significant difference at the $0.001 < p \leq 0.01$ level.

Table S2: Quantitative PCR primer sequences for key *PT1s* in coconut

Gene names	Primer sequences
AZ11G0219160	F:CTTCGGTCCCAACAGCACCA
	R:CCAAGTACAGAAACCCGAACGAG
AZ02G0034860	F:AGAACCAGGACAAGGCTAAGG
	R:AAGTTGTTTCGGATGTGGGATCA
CnActin	F:ATAAAGTATGGCTGATGCTGAGG
	R:CAACAATGCTTGGGAACACA
